# Fragmented QRS as an early predictor of left ventricular systolic dysfunction in healthy individuals: a nested case-control study in the era of speckle tracking echocardiography

**DOI:** 10.1186/s12947-020-00216-z

**Published:** 2020-08-13

**Authors:** Mohammad Hossein Nikoo, Zahra Jamali, Iman Razeghian-Jahromi, Mehrab Sayadi, Paolo Verdecchia, Firoozeh Abtahi

**Affiliations:** 1 Cardiovascular Research Center, Shiraz University of Medical Sciences, Shiraz, Iran; 2 Non-Communicable Diseases Research Center, Shiraz University of Medical Sciences, Shiraz, Iran; 3 Struttura Complessa di Cardiologia, Ospedale S. Maria della Misericordia, S. Andrea delle Fratte, Perugia, Italy

**Keywords:** Fragmented QRS, Global longitudinal strain, Left ventricular dysfunction, Healthy subjects

## Abstract

**Background:**

Several studies addressed the association between fragmented QRS (fQRS) on 12-lead EKG and left ventricular (LV) dysfunction in patients with a variety of cardiovascular disorders. We tested such association in healthy individuals.

**Methods:**

Out of 500 healthy participants without -overt cardiovascular disease from the Shiraz Heart Study cohort, we identified 20 subjects with fQRS (cases) and 20 peers without fQRS (controls). Global LV longitudinal strain (GLS) was measured by speckle tracking echocardiography in the two groups. Comparison was made between case and control groups by using chi-square or independent sample t-test or ANOVA.

**Results:**

Age, gender, ejection fraction, LV volume and dimensions did not differ between the case and the control groups. Overall, 14 subjects out of 40 had reduced GLS (≤20%) and 10 of them (25%) had fQRS. GLS was significantly lower in the group with fQRS than in the control group (19.9 ± 1.8 vs 21.4 ± 1.6; *p* = 0.009).

**Conclusions:**

Healthy subjects with fQRS present regional LV systolic dysfunction, assessed by GLS, in the presence of a normal ejection fraction. These data suggest that fQRS may be a promising tool to identify apparently healthy subjects with regional LV systolic dysfunction.

## Background

Left ventricular (LV) systolic function is an important clinical finding in cardiology. It is applied in prevention, diagnosis, prognostication, and treatment ina variety of cardiovascular conditions. Speckle tracking echocardiography (STE) measures LV systolic function quantitatively through detecting subtle myocardial deformations. Using this technique, the most sensitive and reproducible parameter capable of early detection of malfunctions is the global longitudinal strain (GLS) [1]. The functionality of GLS is more pronounced in the case of left ventricular ejection fraction (LVEF) being normal [2].

Less than two decades ago, fragmentation of QRS complex (fQRS) was coined on 12-lead EKG [3]. Abnormal deflections in QRS morphology is simply known as fQRS which originates from conduction delay and disrupted ventricular depolarization due to myocardial scarring [4, 5]. fQRS is found in several cardiovascular and non-cardiovascular disorders including structural heart diseases [6–8]. Several studies suggested the potential clinical utility of Fqrs. For example, although pathologic Q-wave is known as the marker of myocardial infarction (MI) on a 12-lead EKG, its capacity in detecting myocardial scars is confined to only about one third of patients with documented MI [9, 10]. It has been suggested that fQRS is more sensitive than Q-wave for identifying myocardial scars [11]. Also, an association between fQRS and regional and global LV dysfunction has been reported in patients with coronary artery disease (CAD). Adverse major cardiac events have been predicted by fQRS in these patients in spite of a normal ejection fraction [12].

About 6–10% of apparent healthy individuals show fQRS [13]. In a general population free of clinical cardiac diseases, fQRS was a common finding [14]. Because the majority of studies fQRS was focused on diseased populations, the present study was designed to investigate the capacity of fQRS as an early predictor of LV systolic dysfunction in apparent healthy individuals.

## Methods

This nested case-control study was done in the setting of a prospective cohort, Shiraz Heart Study (SHS), which is conducted on general population of Shiraz city aiming to analyze cardiovascular risk factors [15]. The present study has been conducted in accordance with the declaration of Helsinki and has been approved by the Ethical Committee of Shiraz University of Medical Sciences. All the study subjects provided written informed consent.

Inclusion criteria were negative past medical history, normal lipid profile, normal blood pressure, normal anthropometric indices as well as non-smokers and non-diabetics. Exclusion criteria were history of CAD, history of major risk factors for CAD (hypertension, diabetes mellitus, and hyperlipidemia), angina pectoris, acute coronary syndrome, cardiomyopathies, receiving any cardiovascular-related medications, implantation of pacemaker, heart valve disease, atrial fibrillation and flutter, rheumatism, renal disease, malignancy, pulmonary hypertension, and chronic obstructive pulmonary disease. Among those who met inclusion and exclusion criteria, random sampling was utilized in order to select 500 subjects.

A resting 12-lead EKG has obtained previously from the all the participants as the cohort scheduled procedure (filter settings: 0.5–150 Hz, 25 mm/s, 10 mm/mV). The EKGs of 500 subjects were thoroughly evaluated by two independent cardiologists seeking for QRS fragmentation. Notching in the R or S wave in the absence of a branch block, or an RSR’ pattern additional to the original QRS wave (< 120 ms) were defined as fQRS [16]. Upon disagreement on interpretation of an EKG, it was referred to a third cardiologist. Existence of fQRS was confirmed in conference in twenty subjects (case group). Similarly, out of remaining480, twenty age-matched subjects without fQRS were assigned as control group. Fragmentation was classified based on its location to anterior (V1 to V5), inferior (DII, DIII, aVF), or lateral (DI, aVL, V5, V6) leads.

Subjects in the two groups were asked to attend in the clinic. EKGs were repeated in order to find any possible new changes or arrhythmias by an expert who was blinded to grouping. Then, STE was performed with a commercially available ultrasound scanner (Vivid E9, General Electric Medical Systems, Horten, Norway) with a 2.5-MHz transducer by a single blinded echoman cardiologist. Echocardiograms were obtained in three-, two- and four-chamber apical views at a rate of 50 to 70 frames/s with the patient holding their breath during at least three cardiac cycles. Endocardial borders were automatically marked and tracking was applied to each image. In satisfactory tracking, the entire cardiac wall (endocardium through myoepicardial border) was covered. The LV was divided into four segments in 3-chamber view, and six segments in 2- and 4-chamber view, totally 16 segments were assessed. If the segments were marked by the software automatically, the obtained data were recorded. Otherwise, they were corrected manually. Image analysis was done by AFI system.

Peak systolic longitudinal strains (LS) of different segments were calculated and then, average LS for each view was produced. GLS was the arithmetic mean of LSs in three apical views. GLS of > 20% was assumed to be normal [17]. Dimensions and volumes of the left ventricle were measured according to the guidelines of the American Society of Echocardiography. Also, LVEF was calculated by Simpson rule [18]. Preserved EF was considered as EF ≥50% [19].

The statistical analysis was done in SPSS for Windows (release 14.0, SPSS Inc., Chicago, Illinois). Categorical variables are expressed as number (percentages) and continuous variables as mean ± standard deviation (SD). Comparison between variables were done using chi square, independent samples *t* test or ANOVA when appropriate. *P* value of less than 0.05 was considered statistically significant.

## Results

The age range of the participants was 40 to 60 years old (mean of 50.3 ± 6.5) and higher prevalence of male subjects (75%). (Table 1). Mean LVEF was 59.3 ± 2.9% and the mean GLS was 20.7 ± 1.8 which were within the normal range [17, 19]. Participants were grouped into those with (cases) and without (controls) fQRS (20 subjects in each group). According to the Table 2, there were no significant differences in age, gender, EF, left ventricular end-diastolic volume (LVEDV), left ventricular end-systolic volume (LVESV), and LV size between case and control groups.

**Table 1:** Baseline characteristics of the participants

Variables	Total
Age	50.35 ± 6.54
Gender (male %)	30 (75.0)
LVEDV (ml)	81.25 ± 22.45
LVESV (ml)	34.02 ± 9.12
(D-LV diameter) (cm)	4.66 ± 0.52
(S-LV diameter)(cm)	3.04 ± 0.42
EF (%)	59.30 ± 2.89

Data were presented as mean ± sd or n (%) or (%). *LVEDV* left ventricular end-diastolic volume, *LVESV* left ventricular end-systolic volume, *D-LV* diameter left ventricular diameter in diastole, *S-LV* diameter left ventricular diameter in systole, *EF* ejection fraction

**Table 2:** Baseline characteristics of the participants based on fQRS and GLS status

	fQRS			GLS		
	- (*n* = 20)	+ (*n* = 20)	*P* value	Normal (*n* = 26)	reduced (*n* = 14)	*p* value
Age (yrs.)	49.55 ± 6.51	51.15 ± 6.64	0.446	49.65 ± 7.25	51.64 ± 4.93	0.366
Gender (male, %)	16 (80)	14 (70)	0.465	18 (69.2)	12 (85.7)	0.251
LVEDV (ml)	83.15 ± 24.85	79.35 ± 20.25	0.599	82.80 ± 23.79	78.35 ± 20.25	0.557
LVESV (ml)	35.20 ± 9.87	32.85 ± 8.41	0.423	34.38 ± 9.88	33.35 ± 7.82	0.739
D-LV diameter(cm)	4.69 ± 0.48	4.65 ± 0.59	0.792	4.65 ± 0.58	4.70 ± 0.43	0.780
S-LV diameter (cm)	3.08 ± 0.41	3.01 ± 0.45	0.612	3.06 ± 0.46	3.01 ± 0.37	0.744
EF (%)	59.20 ± 2.53	59.40 ± 3.2	0.830	59.23 ± 2.80	59.42 ± 3.15	0.840

Data were presented as mean ± sd or n (%) or (%). Normal GLS: > 20%. Reduced GLS: ≤20%. *LVEDV* left ventricular end-diastolic volume, *LVESV* left ventricular end-systolic volume, *D-LV* diameter left ventricular diameter in diastole, *S-LV* diameter left ventricular diameter in systole, *EF* ejection fraction

GLS was below the normal range (≤20%) in 14 subjects and 10 of these subjects had fQRS. EF and other variables did not differ significantly between those with reduced GLS and peers with normal GLS.

In the group with fQRS, this was localized in the inferior leads in 50% of subjects, equally distributed in anterior and lateral leads in the remaining subjects. The position of fragmentation was not significantly associateed with abnormal changes in GLS or EF (Table 3).

**Table 3:** EF and GLS of the case group based on fQRS position

Fragmentation				*P* value^a^
	Anterior (25%)	Lateral (25%)	Inferior (50%)	
GLS (%)	20.30 ± 0.51	19.60 ± 1.88	19.94 ± 2.21	0.841
EF (%)	61.40 ± 3.50	56.80 ± 1.78	59.70 ± 3.12	0.071

Data were presented as mean ± sd, ^a^Extracted from ANOVA. *GLS* global longitudinal strain, *EF* ejection fraction

When analyzing the LS in different segments, the group with fQRS showed a lower LS than the control group in five segments which included base-, mid-, and apex- of septal, base of anteroseptal and base of inferior. Also, there was significant reduction of LS in apical 4-chamber view, while LS in apical three- and two-chamber views did not differ significantly between case and control groups. Overall, GLS was significantly lower in the group with fQRS than the control group (Table 4). Moreover, longitudinal strains in different locations of QRS fragmentation (anterior, inferior, and lateral) was compared in Table 5.

**Table 4:** Comparison of echocardiographic parameters between case and control groups

	fQRS			*P* value
	–		+	
ANTSEPT (%)	Base	17.20 ± 2.69	14.85 ± 3.90	**0.032**
	Mid	20.25 ± 3.54	18.15 ± 3.96	0.085
POST (%)	Base	19.45 ± 2.28	18.90 ± 2.61	0.483
	Mid	20.80 ± 2.31	20.20 ± 2.59	0.444
ANT (%)	Base	17.10 ± 2.65	17.40 ± 3.07	0.743
	Mid	20.35 ± 2.96	20.25 ± 3.82	0.927
	Apex	25.60 ± 3.55	23.90 ± 4.27	0.179
INF (%)	Base	19.00 ± 1.78	17.25 ± 2.02	**0.006**
	Mid	21.15 ± 2.08	20.00 ± 2.60	0.131
	Apex	25.85 ± 2.70	24.35 ± 4.13	0.182
LAT (%)	Base	18.95 ± 2.67	17.35 ± 3.54	0.115
	Mid	20.20 ± 2.61	19.55 ± 3.89	0.538
	Apex	24.60 ± 3.91	23.55 ± 4.26	0.422
SEPT (%)	Base	17.15 ± 1.93	15.35 ± 2.06	**0.007**
	Mid	20.80 ± 1.77	18.65 ± 2.37	**0.002**
	Apex	25.90 ± 3.13	23.25 ± 3.46	**0.015**
A3C_GLS (%)		21.07 ± 2.28	19.44 ± 2.92	0.057
A2C_GLS (%)		21.67 ± 1.95	20.74 ± 1.96	0.143
A4C_GLS (%)		21.44 ± 1.81	19.86 ± 2.58	**0.031**
GLS (%)		19.94 ± 1.78	21.41 ± 1.59	**0.009**

ANTSEPT: anteroseptal; POST: posterior; ANT: anterior; INF: inferior; LAT: lateral; SEPT: septal; A3C_GLS: GLS in apical three-chamber view; A2C_GLS: GLS in apical two-chamber view; A4C_GLS: GLS in apical four-chamber view. Bold values imply statistical significance

**Table 5:** Comparison of longitudinal strain in different locations of QRS fragmentation

Variables		fQRS location			*P* value
		Anterior	Inferior	Lateral	
ANTSEPT (%)	Base	16.40 ± 3.84	14.40 ± 3.20	14.20 ± 5.49	0.613
	Mid	19.40 ± 3.04	17.1 ± 4.58	19.00 ± 3.53	0.515
POST (%)	Base	20.0 ± 2.0	18.40 ± 3.06	18.8 ± 2.28	0.558
	Mid	21.6 ± 2.7	19.4 ± 2.22	20.4 ± 3.04	0.309
ANT (%)	Base	16.6 ± 1.14	17.60 ± 3.13	17.80 ± 4.49	0.809
	Mid	22.20 ± 2.16	20.10 ± 4.01	18.60 ± 4.50	0.343
	Apex	26.40 ± 2.88	23.30 ± 3.97	22.60 ± 5.63	0.321
INF (%)	Base	18.40 ± 1.94	16.50 ± 2.12	17.60 ± 1.51	0.215
	Mid	21.80 ± 1.30	19.20 ± 2.78	19.80 ± 2.68	0.189
	Apex	24.80 ± 3.7	24.20 ± 2.89	24.20 ± 6.94	0.965
LAT (%)	Base	16.40 ± 3.91	19.90 ± 3.81	17.20 ± 3.11	0.758
	Mid	17.60 ± 3.78	21.00 ± 3.52	18.60 ± 4.27	0.238
	Apex	21.80 ± 4.65	24.80 ± 4.04	22.80 ± 4.38	0.417
SEPT (%)	Base	16.40	15.20 ± 2.34	14.60 ± 1.81	0.385
	Mid	19.80 ± 2.58	18.40 ± 2.50	18.0 ± 1.87	0.458
	Apex	23.20 ± 2.28	23.60 ± 4.03	22.60 ± 3.78	0.127
A3C_GLS (%)		20.68 ± 1.39	18.67 ± 3.35	19.74 ± 3.11	0.462
A2C_GLS (%)		21.84 ± 0.88	20.43 ± 2.42	20.26 ± 1.49	0.366
A4C_GLS (%)		19.20 ± 2.22	20.69 ± 2.74	18.84 ± 2.46	0.360
GLS (%)		20.30 ± 0.51	19.94 ± 2.21	19.60 ± 1.88	0.841

ANTSEPT: anteroseptal; POST: posterior; ANT: anterior; INF: inferior; LAT: lateral; SEPT: septal; A3C_GLS: GLS in apical three-chamber view; A2C_GLS: GLS in apical two-chamber view; A4C_GLS: GLS in apical four-chamber view

## Discussion

This nested case-control study was designed in order to investigate the association, if any, between fQRS and left ventricular dysfunction in apparently healthy people. Although the correlation between fQRS and cardiac disorders has been demonstrated in several diseased status [6, 20], but the importance of this QRS alteration has never been tested in a general population sample. The main finding of the present study is that in apparent healthy subjects with normal EF, those with fQRS had lower GLS than those without fQRS.

Scarring of the myocardium following by zigzag pattern of electrical conduction produces fQRS spikes [21]. fQRS is known as an indicator of previous myocardial injury and warns possible future adverse cardiac events [21]. It was reported that fQRS possibly is the only evidence of silent MI in high risk individuals [3]. Moreover, fQRS was known as a sign of premature ventricular contractions in individuals without obvious structural heart diseases [22]. It was shown to be superior than Q wave for detecting myocardial scar in terms of sensitivity and negative predictive value, but not of specificity [23]. However, in a more recent study, higher sensitivity and specificity of fQRS than Q wave was declared [24]. Also, in case of disappearance of MI-related Q wave due to revascularization therapies, fQRS would be a validated replacement [20].

Existence of fQRS in different EKG leads simply translates into tissue scarring in different segments of the heart and is associated to the higher incidence of cardiac death and hospitalization [21]. Severity and complexity of CAD was reported to be in relation with the number of EKG leads with fQRS [25]. Accordingly, fQRS could be a guiding tool to identify regions of interest for ablation, being potentially more prone to ventricular arrhythmias [20]. The potential of fQRS in predicting arrhythmic events, need for revascularization, MI, cardiac death, and all-cause mortality was shown in subjects with different cardiac disorders [23, 26, 27]. However, there are reports which questioned the capability of fQRS to localize myocardial scar and predict arrhythmic events, and mortality [4, 28–33].

EF, which is a popular tool to estimate LV function, is able to reflect moderate to severe impairment in the ventricles. Also, this parameter suffers several limitations. Of note, EF mostly contributes to the myocardial changes in radial axis while longitudinal deformations are being neglected [34]. Strain is the more developed and accurate measurement than volumetric parameter of EF. It demonstrates fine myocardial deformations in longitudinal, circumferential, and radial axis and also, changes in torsion [34]. Among strains, GLS is of particular importance due to its sensitivity and robustness [35, 36]. The association of mortality with GLS was stronger than LVEF [37]. GLS, which is obtained by STE, measures myocardial deformations via tracing of speckles’ displacement [5, 16, 38]. Reduction in absolute GLS value is an indicator of a myocardial disease in most cases and portends future adverse events [35].

In an investigation on patients with systemic sclerosis, fQRS was present while LVEF and LV dimensions were normal. Importantly, GLS was significantly lower in these patients than in the control group [39]. In a comparison within apparent healthy individuals, GLS was significantly lower in those with fQRS than those without fQRS despite normal similar EF [40].. Although GLS reduction is a sign of LV malfunction, but GLS is also affected by other factors such as age, gender, and ethnicity [41–44]. Also, changes in physiological parameters like heart rate affects GLS in healthy individuals [45]. Hypertension, obesity, dyslipidemia, diabetes and medications were also considered as factors that modifiy GLS value. Vendor-specific disparities and timing of measurements should also be considered in GLS evaluation [35].

fQRS in individuals with normal EF may be due to the existence of myocardial fibrosis of subclinical scale which in turn boasts fQRS sensitivity [22, 24]. EKG remains a convenient, cost-effective, and informative tool. EKG-born fQRS could play a valuable role in identifying individuals among general population who are prone to LV systolic dysfunction and consequent heart failure. A simple EKG has the potential to draw cardiologists’ attention for further assessment of the heart function with more sophisticated tools and parameters such as STE and GLS to find minor, but life-threatening events.

## Conclusions

Because of the rising interest for fQRS in several pathological conditions, we investigated its importance in apparently healthy subjects. In these subjects, fQRS was associated with regional LV systolic dysfunction, assessed by GLS, in the presence of a normal ejection fraction. These data suggest that fQRS should not be considered as an innocent finding in healthy individuals.

## Data Availability

The datasets used and/or analysed during the current study are available from the corresponding author on reasonable request.

## Abbreviations

LV: Left ventricular
STE: Speckle tracking echocardiography
GLS: Global longitudinal strain
LVEF: Left ventricular ejection fraction
fQRS: Fragmentation of QRS complex
CAD: Coronary artery disease
LS: Longitudinal strains
LVEDV: Left ventricular end-diastolic volume
LVESV: Left ventricular end-systolic volume

## References

[1] Bansal M, Kasliwal RR (2013). How do I do it? Speckle-tracking echocardiography. Indian Heart J.

[2] Marwick TH (2013). Methods used for the assessment of LV systolic function: common currency or tower of babel?. Heart..

[3] Das MK, Khan B, Jacob S, Kumar A, Mahenthiran J (2006). Significance of a fragmented QRS complex versus a Q wave in patients with coronary artery disease. Circulation..

[4] Hayashi T, Fukamizu S, Hojo R, Komiyama K, Tanabe Y, Tejima T, et al. Fragmented QRS predicts cardiovascular death of patients with structural heart disease and inducible ventricular tachyarrhythmia. Circ J. 2013;77(12):2889-97. CJ-13-0335. 10.1253/circj.cj-13-0335 23955291

[5] Bayramoğlu A, Taşolar H, Bektaş O, Kaya A, Günaydın ZY (2019). Association between fragmented QRS complexes and left ventricular dysfunction in healthy smokers. Echocardiography..

[6] Das MK, Zipes DP (2009). Fragmented QRS: a predictor of mortality and sudden cardiac death. Heart Rhythm.

[7] Balta S, Demirkol S, Kucuk U, Arslan Z, Unlu M, Demir M (2013). Fragmented QRS in patients with acute myocardial infarction. Heart Lung.

[8] Chatterjee S, Changawala N (2010). Fragmented QRS complex: a novel marker of cardiovascular disease. Clin Cardiol.

[9] Abdulla J, Brendorp B, Torp-Pedersen C (2001). Køber on behalf of the TRACE study group L. does the electrocardiographic presence of Q waves influence the survival of patients with acute myocardial infarction?. Eur Heart J.

[10] Voon W-C, Chen Y-W, Hsu C-C, Lai W-T, Sheu S-H (2004). Q-wave regression after acute myocardial infarction assessed by Tl-201 myocardial perfusion SPECT. J Nucl Cardiol.

[11] Sadeghi R, Dabbagh V-R, Tayyebi M, Zakavi SR, Ayati N (2016). Diagnostic value of fragmented QRS complex in myocardial scar detection: systematic review and meta-analysis of the literature. Kardiologia Polska (Polish Heart Journal).

[12] Yan G-H, Wang M, Yiu K-H, Lau C-P, Zhi G, Lee SW (2012). Subclinical left ventricular dysfunction revealed by circumferential 2D strain imaging in patients with coronary artery disease and fragmented QRS complex. Heart Rhythm.

[13] ÁDB B (2018). From the boundaries of normality to the acknowledgement of a new nosological entity. Rev Port Cardiol.

[14] Tangcharoen T, Wiwatworapan W, Praserkulchai W, Apiyasawat S, Yamwong S, Sritara P (2013). Fragmented QRS on 12-lead EKG is an independent predictor for myocardial scar: a cardiovascular magnetic resonance imaging study. J Cardiovasc Magn Reson.

[15] Zibaeenezhad MJ, Ghaem H, Parsa N, Sayadi M, Askarian M, Kasaei M (2019). Analysing cardiovascular risk factors and related outcomes in a middle-aged to older adults population in Iran: a cohort protocol of the shiraz heart study (SHS). BMJ Open.

[16] Bayramoğlu A, Taşolar H, Bektaş O, Yaman M, Kaya Y, Özbilen M (2017). Association between metabolic syndrome and fragmented QRS complexes: speckle tracking echocardiography study. J Electrocardiol.

[17] Yingchoncharoen T, Agarwal S, Popović ZB, Marwick TH (2013). Normal ranges of left ventricular strain: a meta-analysis. J Am Soc Echocardiogr.

[18] Cheitlin MD, Armstrong WF, Aurigemma GP, Beller GA, Bierman FZ, Davis JL (2003). ACC/AHA/ASE 2003 guideline update for the clinical application of echocardiography: summary article: a report of the American College of Cardiology/American Heart Association task force on practice guidelines (ACC/AHA/ASE Committee to update the 1997 guidelines for the clinical application of echocardiography). J Am Coll Cardiol.

[19] Ponikowski P, Voors A, Anker S, Bueno H, Cleland J, Coats A (2016). Developed with the special contribution of the heart failure association (HFA) of the ESC. Eur J Heart Fail.

[20] Fares H, Heist K, Lavie CJ, Kumbala D, Ventura H, Meadows R (2013). Fragmented QRS complexes—a novel but underutilized electrocardiograhic marker of heart disease. Critical Pathways Cardiol.

[21] Take Y, Morita H (2012). Fragmented QRS: what is the meaning?. Indian Pacing Electrophysiol J.

[22] Temiz A, Gazi E, Altun B, Güngör Ö, Barutçu A, Bekler A (2015). Fragmented QRS is associated with frequency of premature ventricular contractions in patients without overt cardiac disease. Anatolian J Cardiol.

[23] Das MK, Saha C, El Masry H, Peng J, Dandamudi G, Mahenthiran J (2007). Fragmented QRS on a 12-lead ECG: a predictor of mortality and cardiac events in patients with coronary artery disease. Heart Rhythm.

[24] MacAlpin RN (2010). The fragmented QRS: does it really indicate a ventricular abnormality?. J Cardiovasc Med.

[25] Bekler A, Barutçu A, Tenekecioglu E, Altun B, Gazi E, Temiz A (2015). The relationship between fragmented QRS complexes and SYNTAX and Gensini scores in patients with acute coronary syndrome. Kardiologia Polska (Polish Heart Journal).

[26] Maskoun W, Suradi H, Mahenthiran J, Bhakta D, Das M (2007). Fragmented QRS complexes on a 12-lead ECG predict arrhythmic events in patients with ischemic cardiomyopathy who receive an ICD for primary prophylaxis. Heart Rhythm.

[27] Pietrasik G, Goldenberg I, Zdzienicka J, Moss AJ, Zareba W (2007). Prognostic significance of fragmented QRS complex for predicting the risk of recurrent cardiac events in patients with Q-wave myocardial infarction. Am J Cardiol.

[28] Cheema A, Khalid A, Wimmer A, Bartone C, Chow T, Spertus JA (2010). Fragmented QRS and mortality risk in patients with left ventricular dysfunction. Circ Arrhythm Electrophysiol.

[29] Forleo GB, Della Rocca DG, Papavasileiou LP, Panattoni G, Sergi D, Duro L (2011). Predictive value of fragmented QRS in primary prevention implantable cardioverter defibrillator recipients with left ventricular dysfunction. J Cardiovasc Med.

[30] Pietrasik GM, Polonsky S, Moss AJ, Zareba W (2010). Presence of fragmented wide-qrs complex and the risk of death and sudden cardiac death among madit-ii patients with left bundle branch block. J Am Coll Cardiol.

[31] Carey MG, Luisi AJ, Baldwa S, Al-Zaiti S, Veneziano MJ (2010). deKemp RA, et al. the Selvester QRS score is more accurate than Q waves and fragmented QRS complexes using the Mason-Likar configuration in estimating infarct volume in patients with ischemic cardiomyopathy. J Electrocardiol.

[32] Wang DD, Buerkel DM, Corbett JR, Gurm HS (2010). Fragmented QRS complex has poor sensitivity in detecting myocardial scar. Ann Noninvasive Electrocardiol.

[33] Ahn M-S, Kim J-B, Yoo B-S, Lee J-W, Lee JH, Youn YJ (2013). Fragmented QRS complexes are not hallmarks of myocardial injury as detected by cardiac magnetic resonance imaging in patients with acute myocardial infarction. Int J Cardiol.

[34] Nesbitt GC, Mankad S, Oh JK (2009). Strain imaging in echocardiography: methods and clinical applications. Int J Card Imaging.

[35] Collier P, Phelan D, Klein A (2017). A test in context: myocardial strain measured by speckle-tracking echocardiography. J Am Coll Cardiol.

[36] Negishi K, Negishi T, Kurosawa K, Hristova K, Popescu BA, Vinereanu D (2015). Practical guidance in echocardiographic assessment of global longitudinal strain. JACC Cardiovasc Imaging.

[37] Kalam K, Otahal P, Marwick TH (2014). Prognostic implications of global LV dysfunction: a systematic review and meta-analysis of global longitudinal strain and ejection fraction. Heart..

[38] Sengeløv M, Jørgensen PG, Jensen JS, Bruun NE, Olsen FJ, Fritz-Hansen T (2015). Global longitudinal strain is a superior predictor of all-cause mortality in heart failure with reduced ejection fraction. JACC Cardiovasc Imaging.

[39] Tigen K, Sunbul M, Ozen G, Durmus E, Kivrak T, Cincin A (2014). Regional myocardial dysfunction assessed by two-dimensional speckle tracking echocardiography in systemic sclerosis patients with fragmented QRS complexes. J Electrocardiol.

[40] Yaman M, Arslan U, Bayramoglu A, Bektas O, Gunaydin ZY, Kaya A (2018). The presence of fragmented QRS is associated with increased epicardial adipose tissue and subclinical myocardial dysfunction in healthy individuals. Rev Port Cardiol.

[41] Kocabay G, Muraru D, Peluso D, Cucchini U, Mihaila S, Padayattil-Jose S (2014). Normal left ventricular mechanics by two-dimensional speckle-tracking echocardiography. Reference values in healthy adults. Revista Española de Cardiología (English Edition).

[42] Marwick TH, Leano RL, Brown J, Sun J-P, Hoffmann R, Lysyansky P (2009). Myocardial strain measurement with 2-dimensional speckle-tracking echocardiography: definition of normal range. JACC Cardiovasc Imaging.

[43] Takigiku K, Takeuchi M, Izumi C, Yuda S, Sakata K, Ohte N (2012). Normal range of left ventricular 2-dimensional strain. Circ J.

[44] Zghal F, Bougteb H, Réant P, Lafitte S, Roudaut R (2011). Assessing global and regional left ventricular myocardial function in elderly patients using the bidimensional strain method. Echocardiography..

[45] Cifra B, Mertens L, Mirkhani M, Slorach C, Hui W, Manlhiot C (2016). Systolic and diastolic myocardial response to exercise in a healthy pediatric cohort. J Am Soc Echocardiogr.

